# Altered dynamic functional connectivity characteristics in adolescent depression with childhood trauma: a pilot resting-state EEG study

**DOI:** 10.3389/fpsyg.2026.1841572

**Published:** 2026-07-22

**Authors:** Yunshu Zhang, Jie Wang, Yuting Song, Shuaiyu Long, Yi Li, Tingting Hu, Qianqian Zhang, Wenqing Jin, Bing Li, Chaomeng Liu

**Affiliations:** 1 Hebei Provincial Mental Health Center, Baoding, China; 2 The Sixth Clinical Medical College of Hebei University, Baoding, China; 3 Beijing Key Laboratory of Mental Disorders, National Clinical Research Center for Mental Disorders and National Center for Mental Disorders, Beijing Anding Hospital, Capital Medical University, Beijing, China

**Keywords:** adolescent depression, childhood trauma, dynamic functional connectivity, K-means clustering, resting state EEG

## Abstract

**Introduction:**

This exploratory small sample study aimed to investigate dynamic functional connectivity (dFC) abnormalities in adolescent depression associated with childhood trauma through resting-state electroencephalography (EEG), focusing on identifying atypical patterns in key brain networks and their correlation with clinical symptoms.

**Methods:**

Resting-state EEG data were recorded from adolescents diagnosed with depression, encompassing two subtypes: individuals with a history of childhood trauma (CTD) and those without such a history (NCTD). Additionally, data were collected from a matched group of healthy controls (HCs). Childhood trauma was assessed using the Childhood Trauma Questionnaire-Short Form (CTQ-SF). Depression severity was evaluated using the 17-item Hamilton Depression Rating Scale (HAMD-17). dFC was analyzed using the sliding window method and weighted phase-lag index, followed by K-means clustering to identify key connectivity states across frequency bands. Spearman correlation analysis was performed to explore the relationship between dFC matrix characteristics and clinical features in the CTD group.

**Results:**

The final sample comprised 25 individuals in the CTD group, 27 in the NCTD group, and 32 HCs. The CTD group exhibited an earlier onset and more severe depression compared to the NCTD group, with significant differences (*p* < 0.001). In the CTQ-SF assessment, the CTD group scored significantly higher on emotional abuse, emotional neglect, and somatic neglect compared to both the HC and NCTD groups (*p* < 0.001). dFC analysis and K-means clustering revealed three distinct brain states (S1, S2, S3). These brain states correlated with CTQ-SF scores in the CTD group.

**Discussion:**

Resting EEG with dFC analysis and K-means clustering could be biomarkers for identifying adolescent depression linked to childhood trauma, aiding personalized trauma-focused interventions and monitoring treatment efficacy.

## 1 Introduction

In 2021, the global prevalence of depressive disorders among adolescents aged 10–19 years was approximately 2.4%, with major depressive disorder (MDD) accounting for 2.0% (Zhang et al., 2025). According to the Global Burden of Disease Study 2023 (GBD 2023 Mental Disorder Collaborators, 2026), the age-standardized prevalence of mental disorders (12 categories combined) was 14,210.7 per 100,000 population globally in 2023 (GBD 2023 Mental Disorder Collaborators, 2026). The peak burden has shifted to the 15–19 age group, with a disability-adjusted life year (DALY) rate of 2617.3 per 100,000, largely driven by depressive and anxiety disorders. The presence of depressive symptoms during adolescence increases the risk of future functional impairments and psychological disorders (Copeland et al., 2021). Furthermore, depressive symptoms are among the strongest correlates and predictors of suicidal ideation (Horwitz et al., 2017).

The familial environments established during childhood are pivotal in influencing adolescent development. Adolescents’ perceptions and expressions of difficulties are not primarily derived from a sense of social emptiness; rather, their cognitive status plays a significant role in this process (Giannouli, 2018). A comprehensive meta-analysis has demonstrated a strong association between impaired cognitive function in individuals with mood disorders and experiences of childhood trauma. Such trauma can disrupt normative developmental trajectories (Bick and Nelson, 2016). Childhood trauma encompasses both psychological and physical forms, including sexual abuse, emotional abuse, emotional neglect, physical neglect, and physical abuse (Kessler et al., 2010). These experiences can have both immediate and long-lasting effects on mental health, increasing the risk for a range of issues such as antisocial behavior, sleep disturbances, and depression (Danese, 2020; Fava et al., 2023). Any form of childhood trauma more than doubles the likelihood of developing depression in adulthood (Li et al., 2016). Therefore, childhood trauma is considered a key predictor of depressive symptoms. In clinical practice, adolescents with depression often do not disclose their experiences of childhood trauma, preferring to conceal or avoid discussing such events (Wen et al., 2021). Early identification of individuals with a history of childhood trauma can significantly reduce the risk of major adverse outcomes, such as suicide, thereby improving treatment outcomes and facilitating better integration into social environments.

Despite the well-documented adverse effects of childhood trauma on the quality of life and future productivity of adolescents with depression, the neurobiological characteristics associated with depression in this context are only beginning to be elucidated (Nielson et al., 2021). Del Giacco et al. (2024) reported a correlation between increased childhood trauma and heightened depressive symptoms in adulthood (Del Giacco et al., 2024). Childhood trauma exerts profound and long-lasting effects on cognitive and emotional development. A meta-analysis confirmed that childhood trauma is associated with significant deficits in attention, working memory, emotion regulation, and executive function (Fan and Kang, 2025). Furthermore, a study on Alzheimer’s disease indicated that stressful events during early adulthood may promote phosphorylated-tau in later life, while events during early to middle adulthood may have differential effects on brain integrity (Palpatzis et al., 2022). Additionally, enhanced resting-state functional connectivity between the ventral striatum and cingulate cortex during adolescence has been linked to more severe depressive symptoms and greater exposure to childhood trauma (Del Giacco et al., 2024). This disruption in brain network connectivity may serve as a neurobiological risk factor for adolescent depression in the context of childhood trauma. Investigating the relationship between childhood trauma and brain network connectivity in adolescent depression could further clarify individualized risk factors and guide prevention strategies.

Electroencephalography (EEG) is a widely used technique across various fields, including monitoring anesthesia depth, assessing cognitive state transitions, and evaluating mental workload (Sun et al., 2020). EEG records the brain’s electrical activity by detecting potential changes on the scalp, providing exceptional temporal resolution, typically at the millisecond level (de Aguiar Neto and Rosa, 2019). This makes EEG particularly suited for investigating rapid dynamic changes in brain functional networks associated with complex cognitive processes. Resting-state EEG signals, which reflect intrinsic brain activity in the absence of specific tasks, exhibit oscillatory electrical patterns that can be analyzed in terms of frequency, amplitude, spectrum, and phase (Zhang et al., 2023). Such spontaneous brain activity is considered a crucial indicator of cognitive and behavioral processes (Liu et al., 2022). The human brain displays significant temporal dynamics in functional connectivity (dFC) (Kamarajan, 2023; Lurie et al., 2020), enabling precise characterization of interregional interactions (Wu et al., 2022) and correlating with higher-order cognitive functions (Shine et al., 2019).

Our initial PubMed search reveals that most studies on EEG characteristics in adolescent depression view functional connectivity as static. However, the brain is dynamic, constantly adjusting its connectivity (Breakspear, 2017). dFC offers a more detailed analysis of resting-EEG time series than stationary FC by examining specific time points or windows (Coelli et al., 2025). This approach uncovers details often missed in stationary FC and captures recurring FC patterns, crucial for understanding brain organization variability. EEG, due to its superior temporal resolution, is the preferred method for monitoring rapid network modulations and analyzing connectivity dynamics in both resting-state conditions and task-related or time-locked responses (Coelli et al., 2025). dFC analysis provides valuable insights into the dynamic properties of resting-state brain networks, shedding light on their role in cognitive and emotional regulation in depressive disorders (Gu et al., 2020). This approach has shown heightened sensitivity to pathological mechanisms (Preti et al., 2017; Tang et al., 2021).

This study aimed to systematically examine the abnormalities in dFC associated with adolescent depression and childhood trauma through resting-state EEG, identifying atypical patterns within key brain networks and exploring their correlation with clinical symptoms.

## 2 Methodology

### 2.1 Participants

The sample size was determined utilizing G*Power 3.1 software (Faul et al., 2009). This study employs a cross-sectional comparative design involving three groups, with the primary statistical method being a one-way analysis of variance (ANOVA). Based on prior literature (Hu et al., 2023), a medium to large effect size (0.35) was assumed, alongside a significance level (*α*) of 0.05 and a power (1–*β*) of 0.80. Consequently, the required sample size for each group was calculated to be 29 participants, resulting in a total of 87 participants across the three groups. To account for an anticipated attrition rate of approximately 15%, the sample size was increased to 35 participants per group, culminating in a total of 105 participants.

Between December 2023 and March 2024, this study recruited actually 104 participants, divided into three matched cohorts: 34 adolescents diagnosed with depression and a history of childhood trauma (CTD group), 36 adolescents with depression but no history of childhood trauma (NCTD group), and 34 age- and sex-matched healthy controls (HC group). Each participant underwent comprehensive clinical assessments to ensure diagnostic accuracy and provided written informed consent.

Inclusion criteria for participants in the adolescent depression groups were as follows: (1) fulfillment of the DSM-5 diagnostic criteria for depression; (2) a score of 17 or higher on the 17-item Hamilton Depression Rating Scale (HAMD-17) (Zheng et al., 1988); (3) a score of less than 13 on the 32-item Hypomania Checklist (HCL-32), excluding the presence of hypomania (Yang et al., 2011); (4) age between 12 and 18 years and right-handedness; and (5) completion of at least at least 6 years of education. In addition, in order to ensure the representativeness of the sample, we require that all subjects have not taking antipsychotic drugs. Exclusion criteria included: (1) history of schizophrenia, bipolar disorder, or other psychiatric disorders; (2) history of neurological disorders, seizures, or other forms of brain injury; (3) intellectual impairments; (4) prior neuromodulation therapies, such as transcranial magnetic stimulation or electroconvulsive therapy, within the past month; and (5) visual or auditory impairments.

Participants diagnosed with adolescent depression were classified into the CTD and NCTD groups based on their scores on the Childhood Trauma Questionnaire-Short Form (CTQ-SF) (He et al., 2019). The CTD group included individuals whose scores met or exceeded trauma thresholds in any of the CTQ-SF domains: emotional abuse (≥13), emotional neglect (≥15), physical abuse (≥10), physical neglect (≥10), or sexual abuse (≥8) (He et al., 2019). The NCTD group comprised individuals whose scores were below the threshold in all domains. The inclusion criteria for the HC group were: (1) age and sex matched with the patient groups; (2) possession of at least a primary education and right-handedness; (3) completion of all clinical assessments and EEG scans.

The study was approved by the Ethics Committee of the Hebei Provincial Mental Health Center, Baoding, China (approval number: 2202308) and conducted in accordance with the Declaration of Helsinki. Written informed consent was obtained from all participants and their legal guardians at the time of enrollment.

### 2.2 Neuropsychological and behavioral assessment

Childhood trauma was assessed using the CTQ-SF, a self-report tool comprising five subscales (He et al., 2019). There are several compelling reasons for selecting this particular instrument to measure childhood trauma. Firstly, the CTQ-SF has been extensively validated over the past decade, making it one of the most rigorously examined tools for assessing childhood maltreatment globally (Schmidt et al., 2018). Secondly, its design, consisting of only 28 items presented on a Likert-type scale, facilitates ease of administration, thereby enhancing its usability in research settings. Lastly, as the most recent and author-endorsed version of the Childhood Trauma Questionnaire, it has emerged as a leading measure in the contemporary assessment of childhood trauma (Baker and Maiorino, 2010). Participants rated each item on a 5-point Likert scale, with total scores ranging from 25, indicating no history of abuse or neglect, to 125, reflecting extreme levels of abuse or neglect.

Depression severity was measured using the HAMD-17, a tool developed in the late 1950s that has become the most frequently employed observer-rated measure for depression in research (Rush, 2007). The HAMD17 is an adaptation of the original 21-item version of the scale and serves as a multidimensional assessment tool encompassing various clinical features associated with MDD (Bagby et al., 2004; Kennedy, 2008). This scale evaluates affective symptoms such as depressed mood and anxiety, as well as additional symptoms including insomnia, hypochondriasis, and weight loss. Prior research has validated that the HAMD-17 may be particularly suitable for the multidimensional evaluation of stable clinical conditions, thereby aiding in the direction of targeted interventions (Nixon et al., 2020).

The HCL-32 serves as a widely applicable screening tool for identifying hypomanic features and has been validated across numerous languages (Gamma et al., 2013). The HCL-32 is a self-administered questionnaire with 32 yes/no items designed to screen for hypomanic symptoms, considering the individual’s current mental state. It showed good sensitivity (0.80) and specificity (0.51) at a cut-off of 14, in a sample comprising predominantly outpatients with bipolar disorders (BP) and unipolar depression (UP) in Europe (Angst et al., 2005). A Chinese study validated the simplified Chinese version for mood disorder patients, recommending a cut-off of 13 to better distinguish between BP-II and UP in psychiatric settings in China (Yang et al., 2011). This initial screening can subsequently be followed by a comprehensive clinical diagnostic interview for further assessment.

Cognitive function was assessed using the THINC-Integrated Tool (THINC-it) (Zhu et al., 2023), which includes the subjective cognitive function questionnaire, the Patient Deficits Questionnaire, 5-item version (PDQ-5-D), and four objective cognitive tasks: the Choice Reaction Time task (CRT), the 1-back memory task, the Digit Symbol Substitution Test (DSST), and the Trail Making Test-B (TMT-B). These tasks assess attention, working memory, processing speed, and executive function, respectively. THINC-it is a simple, flexible, and fast tool for assessing cognitive function in depression, usable on mobile devices or computers. It takes 10 to 15 min and provides immediate feedback (Harrison et al., 2018). Unlike traditional tests, it may prevent fatigue. The Chinese version has shown similar effectiveness in adolescent depression studies, particularly in executive function and continuous performance tests (Chen et al., 2022; Diao et al., 2022). The data were standardized into Z-scores using the formula: (participant’s score - mean score of the HC group)/standard deviation of the HC group.

### 2.3 EEG acquisition and preprocessing

EEG data were acquired from March to November 2023. All recordings were conducted in a sound-attenuated, electrically shielded room adjacent to the recording control area, separated by a one-way glass window. The ambient temperature of the room was maintained at 22 ± 1 °C, with dim lighting and minimal background noise. Participants were comfortably seated in a reclining chair and instructed to remain awake, relaxed, and with eyes closed during the 5-min resting-state session, to prevent falling asleep and to minimize eye blinks and movements. EEG signals were captured using a 64-channel Quik-cap system (Compumedics Neuroscan), configured according to the 10–05 electrode placement system, with a sampling rate of 1,000 Hz. Electrode impedances were maintained below 5 kΩ. During acquisition, online band-pass filtering (0.5–100 Hz) was applied, and the signals were referenced to the average of the two mastoids. The raw EEG data were preprocessed using MATLAB 2023a (The MathWorks, Natick, MA, United States) in conjunction with the EEGLAB toolbox (Delorme and Makeig, 2004). Preprocessing involved applying a standard electrode template for channel localization, excluding channels exhibiting negligible EEG activity (Wang et al., 2021), and re-referencing the signals to the bilateral mastoids. Additional steps included bandpass filtering (0.1–45 Hz), segmentation into 2-s epochs (Wait et al., 2014), interpolation, and removal of channels with incomplete or corrupted data (Serinagaoglu Dogrusoz et al., 2024). Independent component analysis (ICA) was applied to isolate and remove ocular and muscular artifacts, while preserving components indicative of neural activity. The cleaned signals were then decomposed into six canonical frequency bands: delta (0.5–5 Hz), theta (5–8 Hz), alpha (8–13 Hz), low-beta (13–20 Hz), high-beta (20–35 Hz), and gamma (35–45 Hz) (Korzeczek et al., 2022).

### 2.4 dFC analysis

dFC was analyzed using sliding time windows of 2 s duration and a 1-s step size (Li et al., 2025) to calculate the weighted phase lag index (wPLI) for each pair of channels within each window. Functional connectivity states were then identified through K-means clustering with the Euclidean distance metric. State characteristics, including occurrence frequency, mean dwell time, and number of transitions, were quantified using MATLAB. K-means clustering (Liao et al., 2014), a widely used algorithm in data analysis, determines optimal categorical attributes by evaluating the similarity between data points. The process begins by selecting k initial cluster centroids, calculating the distance between each data point and these k centroids, and assigning each point to the nearest cluster based on the minimum distance principle, with the Euclidean distance metric employed in this study. The mean of each cluster is then computed and set as the new centroid. This iterative process continues until the centroids converge or the maximum number of iterations is reached. In the K-means clustering analysis conducted using DynamicBC, the maximum recommended number of clusters is set at 6. To objectively determine the optimal number of clusters, we conducted a comprehensive analysis over a range of K values from 2 to 6, concurrently calculating the Silhouette score, Calinski-Harabasz index, and Davies-Bouldin index. Each of these indices independently suggested an optimal *K* value of 3. Consequently, we adopted *K* = 3 as the consensus optimal number of clusters. Subsequently, the final clustering was executed using the K-means algorithm with Euclidean distance. During this process, all sliding-window connectivity vectors from all subjects were concatenated into a single matrix and clustered collectively, thereby ensuring consistent state labels across all participants (Li et al., 2025).

### 2.5 Statistical analysis

Descriptive statistics were reported as the median (25th percentile, 75th percentile) for non-normally distributed variables and as the mean ± standard deviation (SD) for normally distributed variables. Categorical variables were presented as frequencies and percentages. The normality of the variables was verified using the Shapiro–Wilk test. Statistical analyses were conducted using SPSS version 24.0 (IBM SPSS Corp., Armonk, NY, United States), with the following methods: (1) One-way ANOVA or the Kruskal-Wallis test was used to compare demographic, psychometric, and dFC metrics across the three groups; (2) *Post hoc* pairwise comparisons between groups were performed using Tukey’s Honestly Significant Difference (Tukey HSD) test or Dunn’s test with Bonferroni correction, depending on the normality of the data; (3) Pearson’s Chi-squared test or Fisher’s exact test was applied to assess differences in categorical variables between groups. Furthermore, the η^2^ index was utilized to quantify the differences between the three groups, with values less than 0.01 indicating a small effect, values between 0.01 and 0.06 representing a medium effect, and values exceeding 0.06 signifying a large effect (Trigo Sánchez and Martínez Cervantes, 2016). Additionally, Cohen’s d was used to assess the differences between two groups (Sockloff, 1978), where a value less than 0.2 indicated a small effect, values ranging from 0.2 to 0.5 denoted a medium effect, and values greater than 0.5 represented a large effect. Spearman correlation analysis was performed to explore the relationship between dFC matrix characteristics and clinical features in the CTD group. To mitigate the risk of Type I errors associated with multiple testing, the Bonferroni correction was employed (Gordi and Khamis, 2004). All tests were two-tailed, with a significance level set at *p* < 0.05.

## 3 Results

### 3.1 Comparison of demographics and depression levels

**Figure 1:**
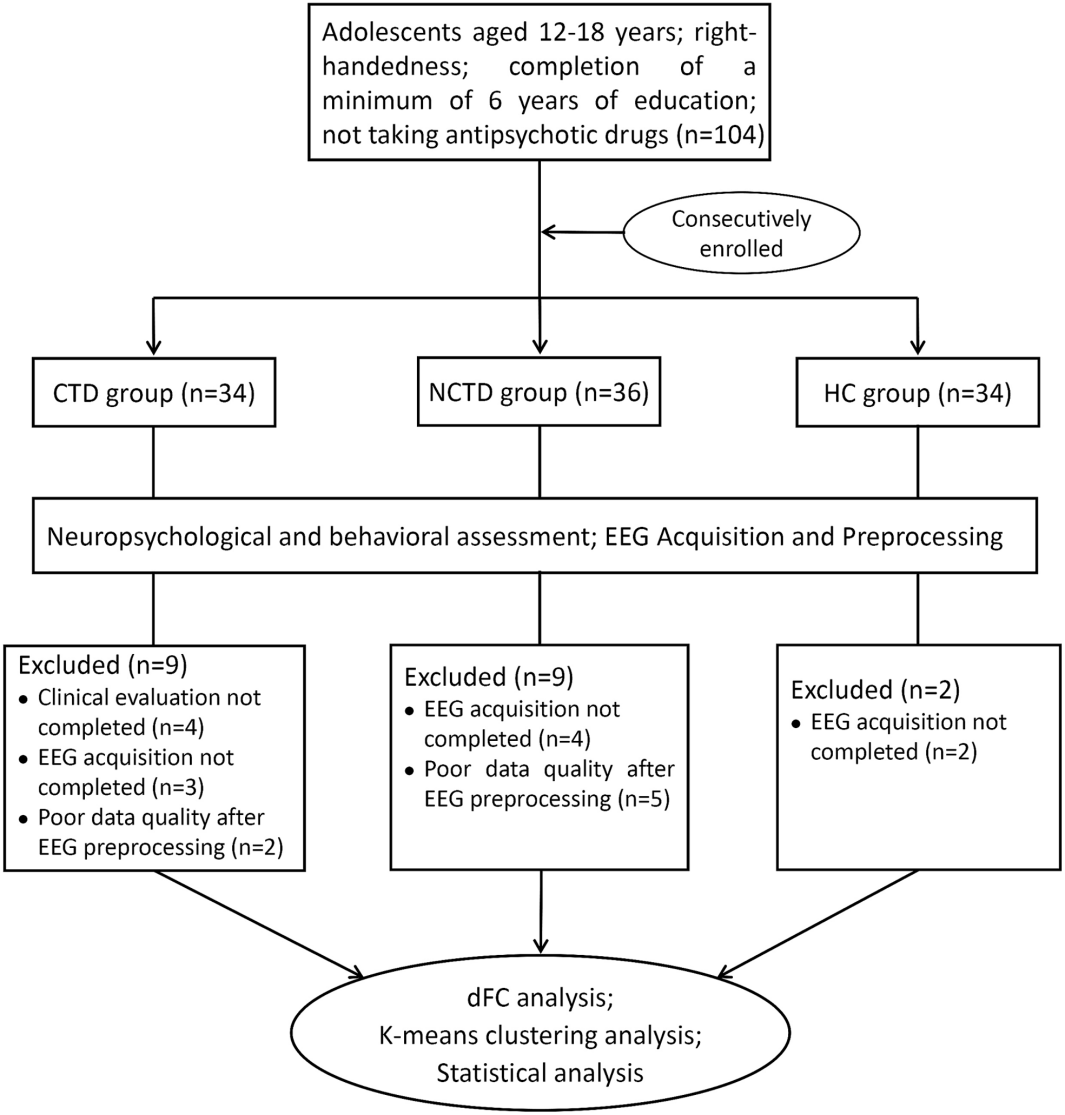
Study flowchart.

In the CTD group, four participants did not complete the clinical evaluation, and three did not complete the EEG signal acquisition. Additionally, during EEG preprocessing, the data quality for two participants was deemed insufficient, resulting in the inclusion of 25 cases in the final statistical analysis. In the NCTD group, four participants failed to complete EEG signal acquisition, and five participants had inadequate EEG data quality during preprocessing. Thus, 27 cases were included in the final analysis. In the HC group, two participants did not complete the EEG signal acquisition, leaving 32 participants for the comparative analysis. The study flowchart is shown in Figure 1.

No significant differences were found among the three groups regarding sex, age, body mass index (BMI), and educational level, with all *p* > 0.05. However, the CTD group had a younger age of depression onset compared to the NCTD group. Furthermore, the CTD group exhibited more severe depression, as reflected in the HAMD-17 scores, compared to the NCTD group, with these differences being statistically significant (all *p* < 0.001), as displayed in Table 1.

**Table 1:** Clinical information of the three groups.

Variables	NCTD group (*n* = 27)	CTD group (*n* = 25)	HC group (*n* = 32)	*z/x^2^*	*p*	*η^2^/Cohen’s d*
Female	20 (74.1%)	19 (76.0%)	17 (53.1%)	4.29	0.117^1^	0.165^1^
Age (years)	15.00 [14.50, 16.00]	15.00 [13.00, 16.00]	16.00 [14.75, 17.00]	5.72	0.057^2^	0.046^1^
BMI	22.04 [18.51, 25.42]	19.49 [18.34, 20.54]	19.96 [18.09, 22.77]	4.76	0.092^2^	0.034^1^
Education (years)	9.00 [8.00, 11.00]	9.00 [8.00, 10.00]	10.00 [8.00, 11.00]	4.11	0.128^2^	0.026^2^
Age of onset	15.00 [14.00, 16.00]	13.00 [12.00, 14.00]	–	−3.56	<0.001^3^	0.716^2^
HAMD-17	20.00 [18.00, 22.50]	23.00 [20.00, 26.00]	–	−2.63	<0.001^3^	0.742^2^

Median (25th percentile, 75th percentile); BMI, body mass index; HAMD-17, 17-item Hamilton Depression Rating Scale; CTD, adolescents with depression and childhood trauma; NCTD, adolescents with depression but no history of childhood trauma; HC, healthy control. ^1^×^2^ test; ^2^Fisher’s Exact Test; ^3^Mann-Whitney *U* test; ^1^η^2^; ^2^Cohen’s d.

### 3.2 Childhood trauma and cognitive performance

**Figure 2:**
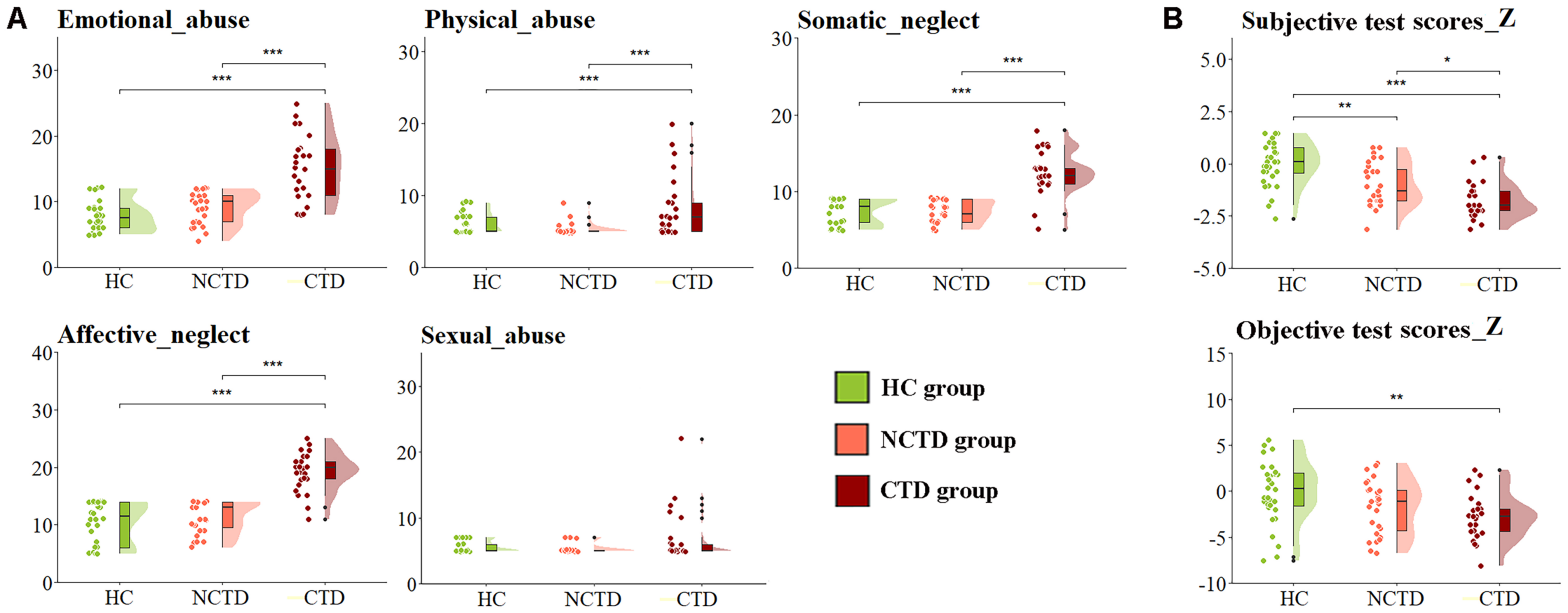
Comparisons of childhood trauma and cognitive function between groups. **(A)** Childhood trauma was compared across the three groups; **(B)** Comparison of cognitive function among the three groups. Childhood trauma was assessed using the Childhood Trauma Questionnaire-Short Form (CTQ-SF), which encompasses five subscales: emotional abuse, emotional neglect, physical abuse, physical neglect, and sexual abuse. Cognitive function was assessed using the THINC-Integrated Tool (THINC-it), which includes the subjective cognitive function questionnaire, the Patient Deficits Questionnaire, 5-item version (PDQ-5-D), and four objective cognitive tasks: the Choice Reaction Time task (CRT), the 1-back memory task, the Digit Symbol Substitution Test (DSST), and the Trail Making Test-B (TMT-B). For ease of comparison, we converted the raw scores of the THINC-it test into Z scores. HC, healthy controls; CTD, depression and a history of childhood trauma; NCTD, depression but no history of childhood trauma. *p < 0.05; **p < 0.01; ***p < 0.001.

In the CTQ-SF assessment, the CTD group demonstrated significantly higher scores on the emotional abuse, physical abuse, emotional neglect, and physical neglect subscales compared to both the NCTD and HC groups, with the differences being statistically significant (*p* < 0.001). Further details are provided in Figure 2A and Supplementary Table 1.

Regarding subjective cognition, as measured by the PDQ-5-D subtest, the NCTD (*p* = 0.009) and CTD groups (*p* < 0.001) exhibited lower standard scores compared to the HC group; additionally, the CTD group scored lower than the NCTD group (*p* = 0.035). In terms of objective cognitive function, the CTD group showed significantly lower standard scores than the HC group (*p* = 0.003), as illustrated in Figure 2B and Supplementary Table 1.

### 3.3 dFC state analyses

**Figure 3:**
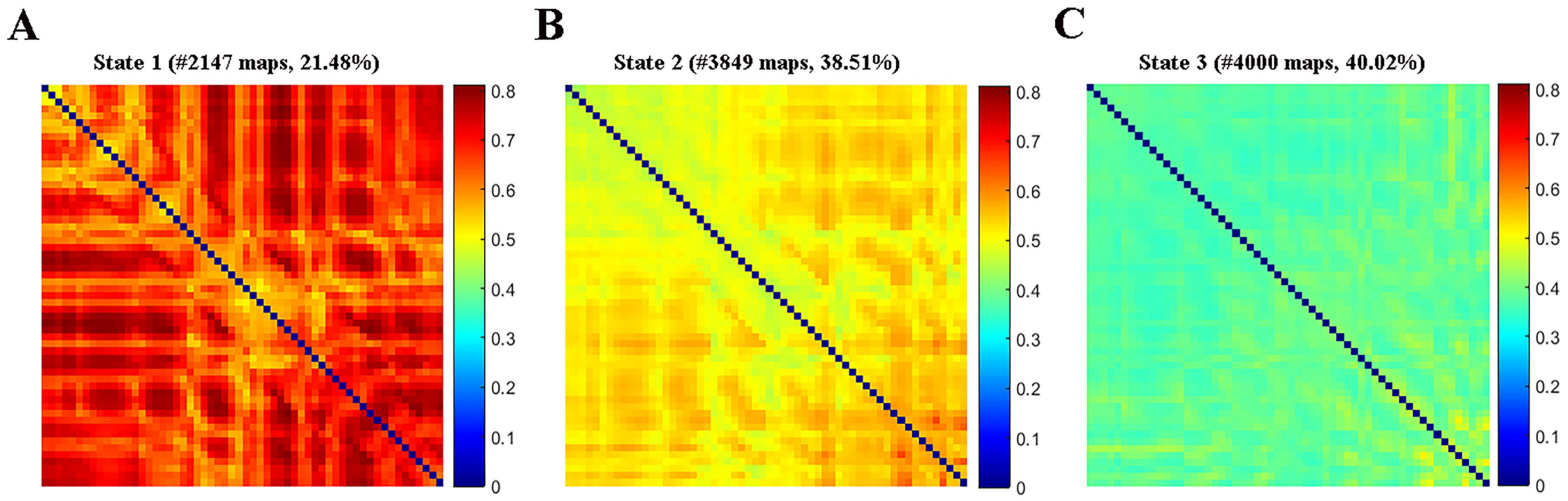
Results of K-means clustering analysis under eyes-closed conditions. **(A)** State 1; **(B)** State 2; **(C)** State 3. The whole brain functional connectivity of all windows of all participants was clustered into three states by K-means clustering method.

The results revealed significant between-group differences in alpha-band dFC, and three characteristic connectivity states (S1, S2, and S3) were identified. An increasing trend in occurrence was observed across the states: S1 (21.48%), S2 (38.51%), and S3 (40.02%). The centroid matrices for these states displayed distinct interregional coupling patterns, providing a basis for subsequent group-level comparisons (Figure 3).

**Figure 4:**
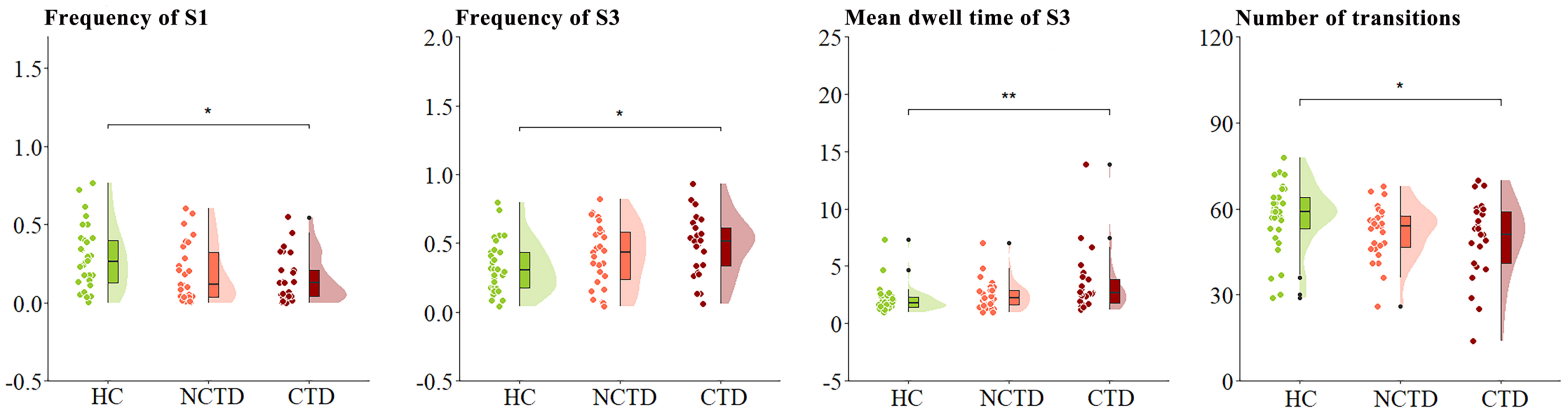
Comparisons of brain state parameters between groups. HC, healthy controls; CTD, depression and a history of childhood trauma; NCTD, depression but no history of childhood trauma; *p < 0.05; **p < 0.01.

Regarding the frequency of occurrence for S1, the CTD group showed a significantly lower frequency than the HC group (*p* = 0.012). In contrast, for the frequency of S3, the CTD group demonstrated a significantly higher frequency than the HC group (*p* = 0.016). The mean dwell time for S3 was also significantly higher in the CTD group compared to the HC group (*p* = 0.003). As for the number of transitions between states, the CTD group exhibited a significantly lower value than the HC group (*p* = 0.013) (Figure 4; Supplementary Table 2).

### 3.4 Correlation analysis

**Figure 5:**
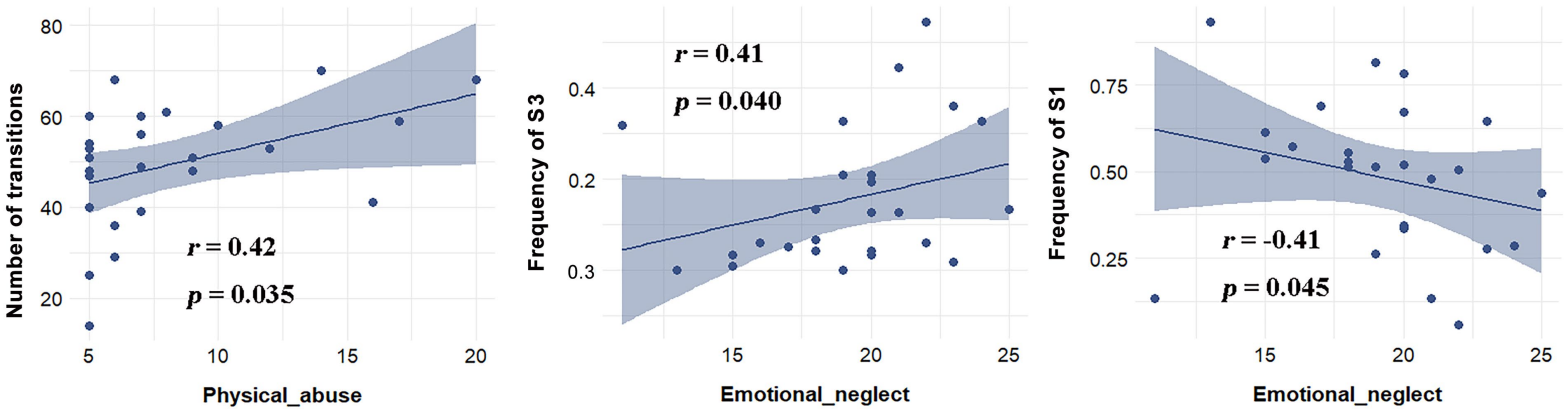
Correlation analysis between statistically significant brain state features and childhood trauma scores in the CTD group.

Correlation analysis revealed a significant association between CTQ-SF subscale scores and dFC features in the CTD group. Scores on the physical abuse subscale were positively correlated with the number of transitions (*r* = 0.42, *p* = 0.035). Additionally, scores on the emotional neglect subscale were positively correlated with the occurrence frequency of S3 (*r* = 0.41, *p* = 0.040) and negatively correlated with the occurrence frequency of S1 (*r* = −0.41, *p* = 0.045) (Figure 5).

## 4 Discussion

This study investigated the characteristics of dFC in adolescent depression associated with childhood trauma, focusing on the temporal dynamics of brain states. The key findings were as follows: (1) The CTD group exhibited an earlier onset of depression and more severe depressive symptoms; (2) dFC analysis and K-means clustering identified three distinct brain states—S1 (21.48%), S2 (38.51%), and S3 (40.02%), with differences in frequency and transitions across groups; (3) These brain states were correlated with CTQ-SF scores in the CTD group.

Adolescents with a history of childhood trauma are typically characterized by earlier onset, higher recurrence, greater comorbidity, more chronic progression, and poorer responses to pharmacotherapy in depressive disorders (Hovens et al., 2010; Klein and Kotov, 2016; Luo et al., 2022; Nanni et al., 2012; Nelson et al., 2017). Previous studies have established a link between early childhood adversity and elevated depression scores, with various forms of childhood trauma exacerbating these outcomes (Humphreys et al., 2020). Our findings further support the view that individuals with childhood trauma experience more severe depressive symptoms. Dysregulated stress response systems and maladaptive personality traits may are among several neurobiological and psychological mechanisms (Kuzminskaite et al., 2021; Lippard and Nemeroff, 2023).

The Chinese simplified version of the THINC-it tool has recently been used in clinical research on adolescent depression (Chen et al., 2022). This tool offers several advantages, particularly its inclusion of both subjective and objective assessments of cognitive function (Harrison et al., 2016). The integration of both assessment types is crucial, as subjective and objective measures often show low correlation but independently contribute to understanding patient functioning (Thomas Gualtieri, 2004). The PDQ-5-D, a subjective cognitive test, which assesses cognitive control functions such as attention, concentration, memory (both prospective and retrospective), planning, and organization, was used in our study (Zhang et al., 2020). The CTD group scored lower than the NCTD group on the PDQ-5-D subtest. Subjective cognitive assessments are more profoundly impacted by the presence and severity of depressive symptoms than objective measures (Lahr et al., 2007; Lehrner et al., 2014). Prior research has shown a correlation between impairments in cognitive control and deficits in cognitive regulation among adolescents, which may exacerbate challenges in sustaining positive social relationships and hinder the ability to shift focus away from negative emotions, such as despair and suffering (Joormann and Quinn, 2014; Wang et al., 2024).

Functional connections across neural networks are increasingly recognized as dynamic, exhibiting temporal fluctuations (Meier et al., 2016). These dFC characteristics reflect transient and recurring whole-brain patterns of temporal coupling, revealing neural mechanisms that are undetectable through traditional resting-state functional connectivity analysis. As a result, an analytical framework combining group ICA, the sliding window approach, and K-means clustering has been proposed to characterize dynamic alterations in disorder-related functional network connectivity (Olivito et al., 2020). In the present study, dFC analysis, coupled with K-means clustering, identified three distinct brain states: S1 (21.48%), S2 (38.51%), and S3 (40.02%). S3, the most frequently observed state among all participants, is characterized by the weakest global connectivity. We interpret S3 as a disconnection pattern dominated by the default-mode network (DMN), indicative of reduced long-range synchronisation and diminished cross-network integration. From a neurobiological perspective, this low-connectivity configuration may result from increased cortical slow-wave activity combined with decreased glutamatergic excitation, potentially reflecting a state of reduced vigilance or drowsiness (Allen et al., 2018). Notably, the CTD group demonstrated a significantly higher frequency and prolonged dwell time in S3 compared to controls, suggesting that childhood trauma predisposes the brain to this hypo-connected mode. This observation is consistent with the hypo-arousal model of trauma-related depression, which posits that chronic early stress downregulates arousal-regulating circuits, particularly the locus coeruleus–noradrenergic system, culminating in a persistent low-energy neural state that manifests phenotypically as anhedonia and emotional blunting (Zhu et al., 2026).

In contrast, S1, although the least frequent, exhibited the most robust interregional coupling. We propose that S1 represents a highly integrated “executive–salience” state, necessitating coordinated activity across frontoparietal, cingulo-opercular, and DMN nodes. This state is critically dependent on dopaminergic D1 receptor-mediated persistent firing within prefrontal circuits (Zheng et al., 2022). The CTD group demonstrated a significantly reduced occurrence of S1 compared to controls, suggesting that trauma impairs the brain’s ability to achieve this metabolically demanding yet functionally optimal configuration. Mechanistically, early adversity may disrupt prefrontal–limbic structural connectivity through impaired myelination and synaptic pruning, as evidenced by diffusion imaging studies in maltreated youth (Zou et al., 2024). The diminished capacity to enter S1 may underlie the executive dysfunction and poor cognitive control frequently observed in trauma-exposed, depressed adolescents. S2, characterized by intermediate connectivity and frequency, likely functions as a transitional or “metastable” state, facilitating the brain’s exploration of alternative network configurations. While our analyses did not reveal significant differences in S2 between groups, its potential role as a neural “gear-shifting” mechanism merits further investigation.

In terms of the frequency of transitions, the CTD group demonstrated a significantly reduced number of state changes compared to the HC group. This observation suggests a pattern of rigid and perseverative neural dynamics, as opposed to chaotic fluctuations. We interpret these findings as indicative of hyper-stability within the DMN and a diminished capacity for network reconfiguration. This pattern aligns with the cognitive inflexibility commonly associated with trauma-related depression (Zhu et al., 2026).

Correlation analysis revealed that higher scores on the physical abuse subscale were associated with increased number of transitions, suggesting that physical abuse may contribute to greater instability in adolescent depression with a history of childhood trauma. Conversely, the emotional neglect subscale score was positively correlated with the occurrence frequency of S3 and negatively correlated with the occurrence frequency of S1, indicating that emotional neglect may be linked to weakened connectivity within the brain’s functional network in the CTD group. It is important to note that while correlations between brain state indices and clinical scale scores were observed within the CTD group, the inclusion of EEG data from HCs during clustering may have introduced heterogeneity into the definition of connectivity states. Future analyses will address this limitation to enhance methodological rigor.

Our findings collectively endorse an integrated dual-pathway model of trauma-related depression in adolescents. This model posits that childhood trauma contributes to both neural stagnation, characterized by prolonged low-connectivity S3 dominance, and state-specific hypo-engagement, indicated by reduced S1 access. Additionally, physical abuse introduces a dynamic instability component. This framework effectively links resting-state neurophysiology with clinical heterogeneity, thereby identifying potential neural targets for trauma-informed interventions (Zou et al., 2024). To substantiate these proposed mechanisms, future research should incorporate a combination of EEG with neurochemical or structural imaging techniques.

### 4.1 Limitations

Several limitations should be considered in this study. First, the small sample size and single-center participant pool may limit the generalizability of the findings to broader populations. Second, we utilized a sliding-window methodology with a window length of 2 s and a step size of 1 s for the analysis of dynamic functional connectivity, adhering to established protocols in the literature. It is important to note, however, that the selection of window parameters can impact the accuracy of temporal estimations and the statistical power of the analysis. Variations in window lengths or step sizes may affect the robustness of the results. Future research could benefit from incorporating data-driven strategies to optimize window parameters, thereby enhancing the validation and generalizability of our findings. Third, although source localization is typically applied before connectivity computation to mitigate confounding effects such as volume conduction and reference electrode bias (Nagy et al., 2024), this study focused on characterizing the temporal dynamics of EEG-derived connectivity patterns, including frequency and dwell time. Future research will incorporate individualized source reconstruction techniques to improve data standardization and spatial accuracy. Fourth, a significant limitation of this study is the heterogeneity present within the childhood trauma group, especially concerning the type, severity, and developmental timing of trauma. Consequently, our findings predominantly illustrate group-level differences and may not be applicable to all subtypes of trauma. Lastly, although the effects of age, sex, and education on dFC were controlled, other confounding variables such as smoking habits (Wootton et al., 2020) and sleep quality may have influenced the results.

## 5 Conclusion

In conclusion, this study identified abnormal dFC characteristics in adolescent depression associated with childhood trauma and their correlation with clinical symptoms, thereby offering multi-dimensional neuroimaging evidence pertinent to clinical practice. Firstly, the characteristics of dFC may serve as potential objective diagnostic markers, addressing the subjective limitations inherent in traditional scales for adolescents and enhancing early and precise identification. Secondly, the correlation between abnormal connectivity and specific trauma symptoms identifies brain circuit targets crucial for the development of individualized treatment strategies, underscoring the importance of trauma-focused interventions. Furthermore, this indicator has the potential to dynamically monitor disease progression and treatment response, thereby serving as an objective biological metric for evaluating therapeutic efficacy. It should be noted that this is a small exploratory study, and the findings should be viewed cautiously.

## Data Availability

The raw data supporting the conclusions of this article will be made available by the authors, without undue reservation.
