# Supplementary material for: Altered dynamic functional connectivity characteristics in adolescent depression with childhood trauma: a pilot resting-state EEG study
Source: Front Psychol. 2026 Jul 22;17:1841572. doi: 10.3389/fpsyg.2026.1841572 (PMC13437390; doi:10.3389/fpsyg.2026.1841572)
Supplement: Supplementary file 1 [file Table_1.DOCX]

Supplementary Information for

**Altered dynamic functional connectivity characteristics in adolescent depression with childhood trauma: A pilot resting-state EEG study**

**Supplementary Table 1**

Significant between-group differences in clinical and cognitive characteristics.

| **Index** | **Comparisons between groups** | ***Z/t*** | ***P*** | ***Cohen's d*** |
| --- | --- | --- | --- | --- |
| Emotional_abuse | CTD - HC | 5.696 | **< 0.001** | 0.621 |
|  | CTD - NCTD | 3.889 | **< 0.001** | 0.424 |
|  | HC - NCTD | -1.687 | 0.275 | 0.184 |
| Physical_abuse | CTD - HC | 1.720 | 0.257 | 0.188 |
|  | CTD - NCTD | 3.884 | **< 0.001** | 0.424 |
|  | HC - NCTD | 2.369 | 0.053 | 0.258 |
| Emotional_neglect | CTD - HC | 6.336 | **< 0.001** | 0.691 |
|  | CTD - NCTD | 5.404 | **< 0.001** | 0.590 |
|  | HC - NCTD | -0.732 | 0.464 | 0.080 |
| Somatic_neglect | CTD - HC | 5.65 | **< 0.001** | 0.616 |
|  | CTD - NCTD | 5.667 | **< 0.001** | 0.618 |
|  | HC - NCTD | 0.247 | 0.805 | 0.027 |
| Subjective/Z-score | CTD - HC | -5.541 | **< 0.001** | 0.676 |
|  | CTD - NCTD | -2.523 | **0.035** | 0.378 |
|  | HC - NCTD | 2.980 | **0.009** | 0.454 |
| Objective/Z-score | CTD - HC | -3.328 | **0.003** | 0.420 |
|  | CTD - NCTD | -1.325 | 0.555 | 0.038 |
|  | HC - NCTD | 1.992 | 0.139 | 0.279 |

**Abbreviation**

CTD: adolescents with depression and childhood trauma; NCTD: adolescents with depression but no history of childhood trauma; HC: healthy control. Cognitive function was assessed using the THINC-Integrated Tool (THINC-it), which includes the subjective cognitive function questionnaire, the Patient Deficits Questionnaire, 5-item version (PDQ-5-D), and four objective cognitive tasks: the Choice Reaction Time task (CRT), the 1-back memory task, the Digit Symbol Substitution Test (DSST), and the Trail Making Test-B (TMT-B). The data were standardized into Z-scores using the formula: (participant's score - mean score of the HC group) / standard deviation of the HC group.

**Supplementary Table 2**

Significant between-group differences in state metrics.

| **Index** | **Comparisons between groups** | **Z/t** | ***P*** | ***Cohen's d*** |
| --- | --- | --- | --- | --- |
| Frequency of S1 | HC/CTD | -2.518 | **0.012** | 0.275 |
| Frequency of S3 | HC/CTD | 4.238 | **0.016** | 0.107 |
| Mean dwell time of S3 | HC/CTD | 2.964 | **0.003** | 0.323 |
| Number of transitions | HC/CTD | -2.473 | **0.013** | 0.270 |

**Abbreviation**

CTD: adolescents with depression and childhood trauma; NCTD: adolescents with depression but no history of childhood trauma; HC: healthy control.
